# Repression of Septin9 and Septin2 suppresses tumor growth of human glioblastoma cells

**DOI:** 10.1038/s41419-018-0547-4

**Published:** 2018-05-03

**Authors:** Dongchao Xu, Ajuan Liu, Xuan Wang, Yidan Chen, Yunyun Shen, Zhou Tan, Mengsheng Qiu

**Affiliations:** 1 Institute of Life Sciences, Key Laboratory of Organ Development and Regeneration of Zhejiang Province, College of Life and Environmental Sciences, Hangzhou Normal University, Zhejiang, 311121 China; 2 Institute of Cognitive Neuroscience and Department of Psychology, Zhejiang Sci-Tech University, Hangzhou, China; 3 Department of Anatomical Sciences and Neurobiology, University of Louisville, Louisville, KY 40292 USA

## Abstract

Glioblastoma (GBM) is the most common primary malignancy of the central nervous system (CNS) with <10% 5-year survival rate. The growth and invasion of GBM cells into normal brain make the resection and treatment difficult. A better understanding of the biology of GBM cells is crucial to the targeted therapies for the disease. In this study, we identified *Septin9* (*SEPT9*) and *Septin2* (*SEPT2*) as GBM-related genes through integrated multi-omics analysis across independent transcriptomic and proteomic studies. Further studies revealed that expression of SEPT9 and SEPT2 was elevated in glioma tissues and cell lines (A172, U87-MG). Knockdown of SEPT9 and SEPT2 in A172/U87-MG was able to inhibit GBM cell proliferation and arrest cell cycle progression in the S phase in a synergistic mechanism. Moreover, suppression of SEPT9 and SEPT2 decreased the GBM cell invasive capability and significantly impaired the growth of glioma xenografts in nude mice. Furthermore, the decrease in GBM cell growth caused by SEPT9 and SEPT2 RNAi appears to involve two parallel signaling pathway including the p53/p21 axis and MEK/ERK activation. Together, our integration of multi-omics analysis has revealed previously unrecognized synergistic role of SEPT9 and SEPT2 in GBM, and provided novel insights into the targeted therapy of GBM.

## Introduction

Glioblastoma (GBM), which starts in the brain and spine with approximately 210,000 new diagnoses per year around the world^1^, account for 81% of primary malignant brain tumors^2^. According to their origins, there are three types of gliomas including astrocytic tumors (World Health Organization classification astrocytoma grades I, II (astrocytoma), III (anaplastic astrocytoma), and IV (GBM)), oligodendrogliomas, ependymomas, and mixed gliomas^3^. Although it has been 90 years since it was termed by Percival Bailey and Harvey Cushing. GBM is still difficult to treat and has a poor prognosis with the median survival of about 1 year among patients^4,5^. Despite the advances in safe resection, radiation therapy, and chemotherapy, the remaining GBM cells generally continue to grow and become drug resistance^6,7^. Thus, more effective and targeted treatment strategies are required based on improved and comprehensive understanding of the molecular pathophysiology of the GBM.

Although the molecular mechanisms remain largely elusive, a large amount of GBM transcriptomic data has been accumulated around the world in the past 10 years^8–10^. Due to its complexity, an adequate description of GBM system requires the combination of various molecular biological data from RNA to protein level^11^. Therefore, multi-omics approach aimed at integrating quantitative data of different biological molecules is necessary for discovery of key GBM molecules that are fundamental to regulate the GBM progression and provide potential targets for GBM treatments. Due to its multiform and gradual drug resistance, it is usually unrealistic that a single target could be enough to treat multifactorial tumors such as GBM^12,13^. Therefore, there is a strong rationale for developing multi-target therapies for GBM^14,15^. In light of these considerations, we applied an unbiased multi-omics method for integrating results from microarray multiplex analysis and proteomic identification analysis. This combinatory approach revealed two novel GBM-related molecules, Septin9 (SEPT9) and Septin2 (SEPT2).

Septins are a family of highly conserved GTP­binding and membrane-interacting proteins from yeast to human^16–18^, which are involved in various cellular processes such as cytoskeleton organization, cytokinesis, and membrane dynamics^19–21^. By now, 13 functional Septin genes (*SEPT1* to *SEPT12* and *SEPT14*) have been identified in human^22^, which can be further divided into four subgroups based on their sequence homologies (*SEPT2*, *SEPT3*, *SEPT6*, *SEPT7* subgroup)^23^. The Septin family members can mutually form apolar tri-, hexa-, or octameric complexes with strong affinity^24^, implying their functional interactions. However, the significance and mechanisms of their interactions are poorly understood. In addition, Septins have also been suggested to participate in a variety of cellular functions such as chromosome segregation, DNA repair, cell polarization, migration, and apoptosis^25–27^.

Currently, numerous studies have reported that mis-regulation of Septin expression or activity is associated with human tumorigenesis^28^. High levels of expression of five Septins (SEPT2, 7, 8, 9, and 11) were detected in breast cancer^29–31^. Among them, *SEPT9* was identified as an oncogene in breast, ovarian, head and neck, prostate, and colorectal cancers^32–35^. SEPT2 downregulation was shown to suppress hepatoma cell growth by PPARγ (Peroxisome proliferator-activated receptor gamma) activation^36^. In this study, we identified *SEPT9* and *SEPT2* as GBM associate genes in our multi-omics analysis, and found that suppression of their expression in GMB cells can repress the pathogenesis and progression of GBM both in vitro and in vivo.

## Materials and methods

### Integrated multi-omics analysis

**Fig. 1:**
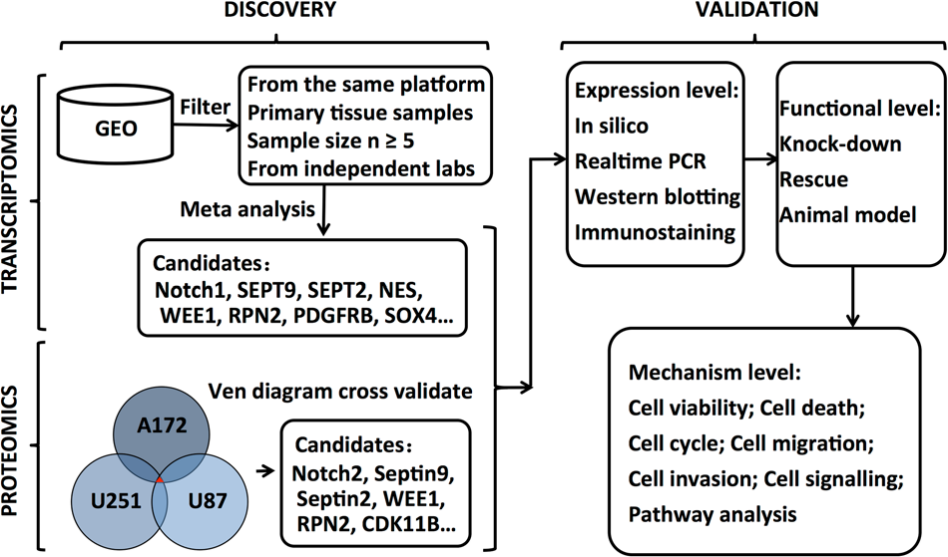
Study outline of integrated multi-omics based discovery and validation of GBM associate genes. The transcriptomic analysis was based on four independent GBM studies, and the proteomic analysis was derived from three different GBM cell lines

Four previous GBM transcriptomic studies^37–40^ were selected based on the following criteria: (1) two types of comparable samples, primary tumor tissues, and normal controls were included; (2) more than five cases vs. controls were used; (3) experiments were run on the same platform (Affymetrix Human Genome U133 Plus 2.0 array); (4) the studies were conducted by independent groups (Supplementary Table S1). In parallel, proteomic profiling of three different GBM cell lines was performed to represent gene expression at protein level. As shown in the sketch of multi-omics analysis workflow (Fig. 1), our study was performed in two phases: (1) the discovery phase, the inputs are the data generated with both proteomic and transcriptomic studies, whereas the output is high-quality functional gene candidates ranked with respect to different statistical criteria; (2) the validation phase, which comprised in silico and experimental evaluation of the gene candidates.

### Antibodies and cell culture

Anti-SEPT9, anti-SEPT2, anti-p53, and anti-p21 antibodies were obtained from Abcam (Abcam, Cambridge, MA, USA). Anti-GAPDH (Glyceraldehyde 3-phosphate dehydrogenase) mouse mAb was obtained from Millipore (Millipore, Hayward, CA, USA). Anti-phospho-MEK1/2 (Mitogen-activated protein kinase kinase 1/2), anti-Erk1/2 (Extracellular signal-regulated kinase), anti-phospho-Erk1/2, anti-Akt (Protein kinase B), and anti-phospho-Akt antibodies were purchased from Cell Signaling Technology (Beverly, MA, USA). Unless specifically stated, all other reagents were commercially purchased.

A172 (CRL-1620), U251, and U87-MG human GBM cells obtained from American Type Culture Collection (ATCC) and Shanghai Cell Bank of the Chinese Academy of Sciences (CAS) were maintained in a humidified incubator at 37 °C in a 5% CO_2_ atmosphere in Dulbecco’s modified Eagle’s medium (DMEM, Gibco, Grand Island, NY, USA) supplemented with 10% fetal bovine serum (FBS, Gibco), and antibiotics (Gibco). Human dermal fibroblast (HDF) cells were primarily derived from the dermis of normal human adult skin and cultured in DMEM with 5% FBS. HEK293T for lentiviral production were purchased from ATCC and cultured in DMEM supplemented with 10% FBS. Cells were passaged three times a week when confluent and only low passage cells (within passage 6) were used.

### Immunocytochemistry and immunohistochemistry

For immunocytochemistry, cells at passage 3–6 were cultured in DMEM with 10% FBS for 24 h. With 50% confluence, cells were fixed in 4% paraformaldehyde (PFA) and blocked with 0.5% BSA (Bovine serum albumin) in PBST (Phosphate Buffered Saline with Tween 20). Cells were incubated with different primary antibodies at 4 °C overnight. Finally, rhodamine or FITC (Fluorescein isothiocyanate)-conjugated secondary antibodies were used for antibody localization and the nuclei were counter-stained with DAPI (4',6-diamidino-2-phenylindole).

To validate the expression of SEPT9 and SEPT2 in gliomas, we analyzed human tissue arrays from 12 malignant GBM (Grade 4), 24 benign brain tumor (Grades 2–3), and 12 normal brain tissue samples, which were purchased from US Biomax Inc. (Rockville, MD, USA). For immunohistochemical staining, antigen retrieval and section staining methods were applied as previously described^41^. Briefly, all samples were washed in xylene to remove the paraffin and then rehydrated through serial dilutions of alcohol. Treated sections were washed with phosphate-buffered saline (PBS) and then heated in a citrate buffer (pH 6.0) for antigen retrieval. The samples were then incubated with anti-Septin antibody for 1 h at 37 °C. The conventional ABC peroxidase method (Vector, Burlingame, CA, USA) was performed for signal development and the cells were counter stained with hematoxylin. Negative controls were obtained by omitting the primary antibody.

### Construction of shRNA-expressing plasmid and viral production

A pCDH-CMV-MCS-EF1-GreenPuro plasmid (SBI System Biosciences, Palo Alto, CA, USA) was used to construct the short hairpin RNA (shRNA)-expressing vector. The sequences listed in Supplementary Table S2 were inserted for SEPT9 and SEPT2 shRNA expression constructs. The viral production started by co-transfecting 293T cells with the shRNA expression vector and the packaging plasmids. After 48–72 h, the media containing the viral particles were harvested and cellular debris was removed from the culture media by centrifugation. The pseudo-viral particles can be precipitated by centrifugation with PEG (Polyethylene glycol) for concentration before added to infect A172 or U87-MG cells.

### Gene expression analysis with qRT-PCR

Total RNA from GBM cells was isolated using Trizol reagent (Thermo Fisher Scientific) for quantitative real-time PCR (qRT-PCR) analysis. Amplification reaction was performed with CFX96 real-time PCR detection system (Bio-Rad, Hercules, CA, USA) using SingleShot SYBR Green qRT-PCR Kit according to the manual (Bio-Rad). The primers for SEPT9 and SEPT2 were listed in Supplementary Table S3 and relative gene expression was calculated using the 2^-ΔΔC^_T_ method. All qRT-PCR experiments were performed in triplicates, and the data were normalized to the expression of GAPDH.

### Western blot analysis

For western blot analysis, proteins were extracted in RIPA buffer (50 mM Tris–HCl, pH 7.4, 1% Triton X-100, 0.25% sodium deoxycholate, 150 mM NaCl, 1 mM EDTA, 0.1% sodium dodecyl sulfate (SDS) and a protease inhibitor cocktail) and separated by SDS-polyacrylamide gel electrophoresis (PAGE). The resolved proteins were transferred to PVDF (Polyvinylidene fluoride) membranes (Millipore). Nonspecific reactivity was blocked by incubating the membrane in 10 mM Tris–HCl (pH 7.5), 150 mM NaCl, 2% Tween 20 and 4% bovine serum albumin 1 h at 37 °C. Diluted primary antibody was then added, followed by the appropriate secondary antibody. Protein detection was achieved with the enhanced chemiluminescence (ECL) system (Thermo Fisher Scientific). Relative protein level was calculated as a percentage of reference protein GAPDH.

### Cell proliferation and methyl thiazolyl tetrazolium (MTT) assay

A172 and U87-MG cells at passage 3–4 were seeded into 96-well plates at 5000 cells per well. After infections with lentivirus as described above, on each day of consecutive 7 days, 10 μL MTT (5 mg/mL) was added to each well and the cells were incubated at 37 °C for additional 4 h. Then, the supernatant was discarded and the reaction was terminated by lysing the cells with 100 μL DMSO (Dimethyl sulfoxide). After 4-h incubation, optical absorption value at 570 nm was measured and the data were presented as the mean ± standard deviation (SD), which were derived from triplicate samples of at least three independent experiments. In parallel, cell growth curve was also plotted with cell counting.

### Synergy determination

The isobologram analysis for double knockdown of SEPT9 and SEPT2 was based upon the Chou-Talalay method to determine combination indices (CIs). The data obtained with the MTT assay were normalized to the Scramble control and expressed as % viability. The data were then converted to Fraction affected (Fa; range 0–1; where Fa = 0 represents 100% viability and Fa = 1 represents 0% viability) and analyzed with the CompuSyn program (Biosoft, Ferguson, MO) based upon the Chou and Talalay median effect principle^42^. The CI values reflect the ways of interaction between SEPT9 and SEPT2 knockdown. CI < 1 indicates synergism, CI = 1 indicates an additive effect, and CI > 1 indicates antagonism.

### Wound-healing motility and transwell invasion assays

Cell migration was determined using a wound-healing assay. A172 or U87-MG cells (1 × 10^6^/mL/well) at passage 3–4 were serum starved for 24 h and then seeded into six-well plates and allowed to adhere for 12 h. Confluent monolayer cells were scratched by a sterile 200-μL pipette tip. The cells were washed with PBS to clear debris and suspension cells. Fresh serum-free medium with different lentiviral treatments were added, and the wounds were observed under a phase contrast microscope at 0 and 24 h. Migration distance was calculated from the change in wound size during 24-h period using Image J software.

Cell invasion ability was measured using a transwell assay. Briefly, A172 or U87-MG cells (5 × 10^4^) at passage 3–4 were suspended in serum-free medium with different lentivirus treatments. Transwell insert chambers (Corning Life Sciences, Corning, NY, USA) with 8-μm pore filters were coated with a final concentration of 0.5 mg/mL Matrigel (BD Sciences, Bedford, MA, USA). Cells were seeded into the top chambers of the wells in 200-μL media, and the lower chambers were filled with 600 μL of 10% FBS media to induce cell invasion. After 24-h incubation, cells on the filter surface were fixed in 4% PFA and examined under a fluorescence microscope, and the numbers of green cells were counted.

### Annexin V-7-AAD apoptosis assay

Cell apoptosis was assessed by 7-aminoactinomycin D (7-AAD) and Annexin V-PE double staining (BD Sciences). The treated cells were collected and washed three times with PBS, and then incubated in 200 μL of staining solution containing Annexin V-PE (Phycoerythrin) antibody and 7-AAD for 15 min in the dark at room temperature. Cells at passage 4–5 were analyzed immediately on an Accuri C6 flow cytometer (Becton Dickinson, Mountain View, CA, USA) using CFlow Plus software. For each measurement, at least 2 × 10^4^ cells were counted and the cell apoptosis rate was determined in three independent experiments.

### Flow cytometric analysis of the cell cycle

Cell cycle analysis was performed using propidium iodide (PI) staining for DNA quantitation. Cells at passage 4–5 were harvested, washed and centrifuged at 1000 r/min for 5 min, and subsequently fixed in 70% ethanol at 4 °C for >1 h, followed by washing with PBS. Cells were then resuspended in 400 μL PBS with 0.05% Triton X-100, 0.1 mg/mL DNase-free RNase A, and 25 μg/mL PI and incubated for 30 min at 37 °C in the dark. For each measurement, at least 2 × 10^4^ cells were analyzed using an Accuri C6 flow cytometer. The cell cycle data were processed using ModFit LT 3.2 (Verity Software House, Topsham, ME, USA).

### Tumor growth assay in vivo

Thirty-five-week-old female immune-deficient nude mice (BALB/c-nu) were purchased from Shanghai SLAC Laboratory Animal Company. The mice were maintained in the facility of laboratory animals, Hangzhou Normal University. The protocol for the experiment was approved, and animals were handled according to the ethical standards of the Institutional Animal Care and Use Committee of Hangzhou Normal University. The mice were assigned randomly to 1 of 5 groups for injection with Control, Scramble, SEPT9-sh, SEPT2-sh and SEPT2, 9-sh U87-MG cells. In all, 2 × 10^6^ cells at passage 5–6 were injected subcutaneously into the right flank of the nude mice^43^, which led to palpable nodules on day 5. The GFP (Green fluorescent protein)-labeled GBM cells were traced with In Vivo Imaging System (PerkinElmer, San Jose, CA, USA), and the tumor volume was measured with calipers every 4 days through the observation period of 3 weeks, using the formula: Volume = length × width^2^ × 0.5^44^. All the mice were sacrificed at day 21 and the tumor weights were measured.

### Statistics

All experiments were performed in triplicate. Data were analyzed by SPSS12.0 and expressed as means ± SD. Statistical comparisons between two groups were made using an unpaired Student’s *t*-test and probability values (*p*) < 0.05 were considered significant.

## Results

### Identification of SEPT9 and 2 as GBM associate genes by multi-omics analysis

To discover GBM associate genes, we combined GBM expression studies from the Gene Expression Omnibus (GEO) repository for a multiplex analysis. For each of the 47,000 transcripts tested, we calculated the Meta fold-change by taking a linear combination of effect sizes (fold-changes) weighted by the variance within each study, and the Meta *p*-values across all studies by using Fisher’s method^45^. Significant genes were selected if the Meta fold-change is >1.5 and the Meta effect *p*-value was <4.5 × 10^−5^. This effort identified *Notch1*, *SEPT9*, *SEPT2*, *NES*, *WEE1*, *RPN2*, *PDGFRB*, *SOX4*, and others as GBM associate genes. We then filtered the candidates through a list of proteins derived from proteomic profiling of three different GBM cell lines, and further narrowed down SEPT9, SEPT2, WEE1, RPN2, and others as the final candidates (Fig. 1). We chose SEPT9 and SEPT2 for further validation as Septins have been implicated in cell proliferation, migration, and tumorgenesis but their roles in GBM have not been determined.

### Expression of SEPT9 and SEPT2 in GBM tissues and cell lines

**Fig. 2:**
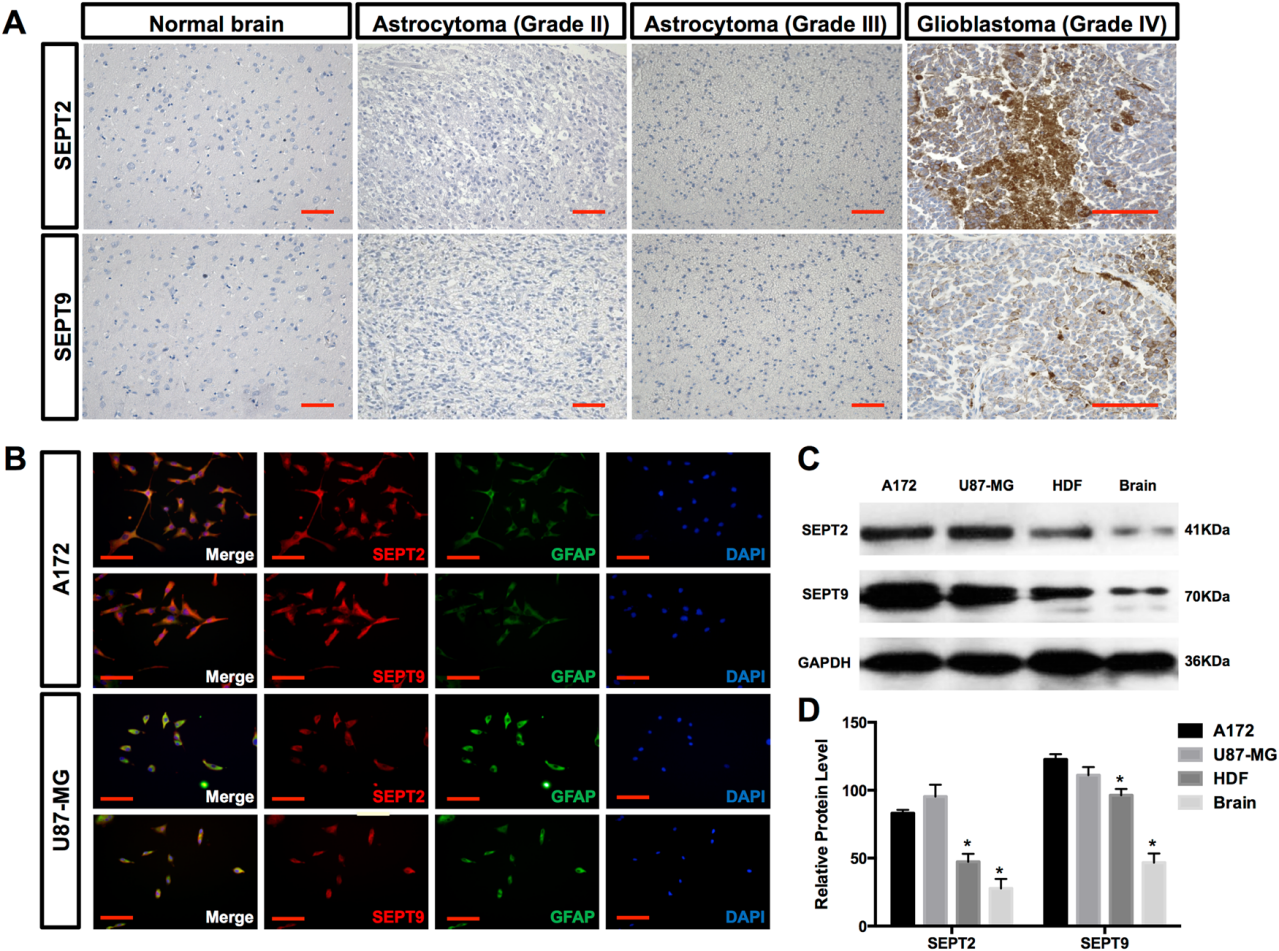
Expression pattern of SEPT2 and SEPT9 in normal and glioma tissues and GBM cell lines. **a** The expression levels of SEPT2 and SEPT9 in 3 grades of gliomas and 12 normal brain tissues were detected by immunohistochemistry. SEPT2 and SEPT9 expression levels were much higher in GBM tissues than in the normal brain tissues, scale bar = 100 μm. **b** A172 and U87-MG cells stained for SEPT2 and SEPT9 showed a perinuclear as well as membrane-bound immunoreactivity colocalizing with GFAP, scale bar = 50 μm. **c** SEPT2 and SEPT9 expression levels in A172 and U87-MG are greater than that in HDF and normal brain tissues. **d** Quantitative analysis of the relative protein levels of SEPT2 and SEPT9 (percentage of GAPDH) from western blot (*p < 0.05)

To gain insight into the role of SEPT9 and SEPT2 in GBM, we first examined our multi-omics mining results in Oncomine database. The upregulated mRNA levels of SEPT9 and SEPT2 in GBM were validated in three independent studies (Supplementary Fig. S1A and S1B). In order to determine the potential clinical relevance of *SEPT9* and *SEPT2* genes, we analyzed TCGA RNA-Seq data set of GBM and found that their expression levels were significantly associated with unfavorable survival in patients with GBM (Fig. S1C and S1D). We further analyzed the expression of SEPT9 and SEPT2 in normal brain tissues (*n* = 12); low-grade glioma tissue samples (grade 2, astrocytoma: *n* = 8); grade 3 glioma samples (anaplastic astrocytoma: *n* = 12), and grade 4, GBM (*n* = 12). Immunohistochemical analysis revealed the increased expression of SEPT9 and SEPT2 in grade 4 GBM tissues (Fig. 2a). The immunocytochemical and western blot analysis demonstrated increased expression levels of SEPT9 and SEPT2 in GBM cell lines (Fig. 2b), as compared with normal brain and HDF cells (Figs. 2c, d). Thus, SEPT9 and SEPT2 expression is upregulated in high-grade GBM tissues, as well as in several GBM-derived cell lines, such as A172 and U87-MG.

### Suppression of SEPT9 and SEPT2 expression using shRNA in A172 cells

**Fig. 3:**
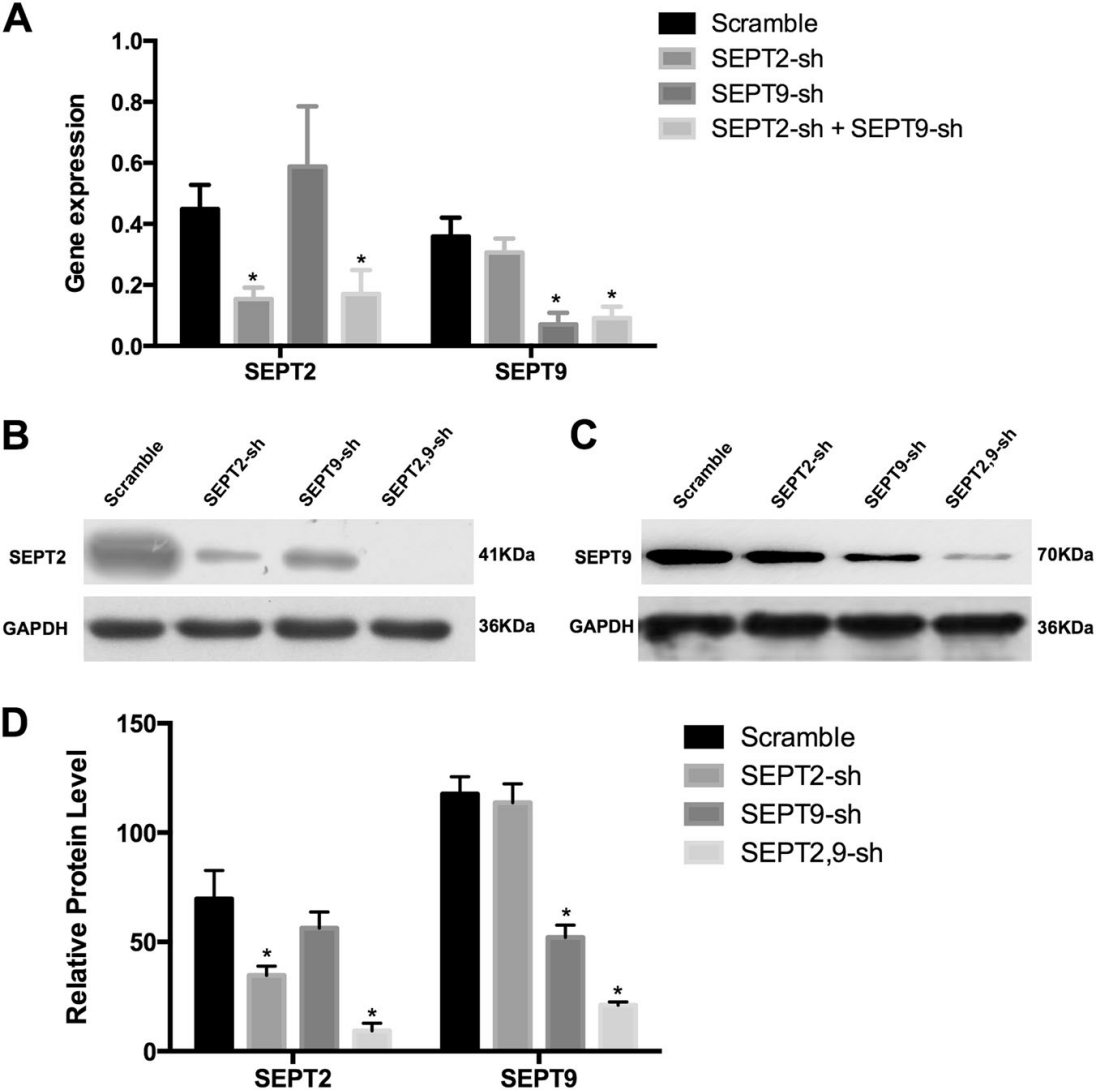
Knocking-down of SEPT2 and SEPT9 mRNA reduced their protein levels. The efficacy of shRNA mediated stable knockdown against SEPT2 and SEPT9 mRNA **a** was verified by western blot with the protein levels in A172 cells. **b**, **c** The downregulated SEPT9 could slightly reduce the protein level of SEPT2, and vice versa. **d** Quantitative analysis of SEPT2 and SEPT9 levels relative to GAPDH as percentage from western blot (*p < 0.05)

To study the roles of SEPT9 and SEPT2 in GBM, we selected two shRNA sequences each for SEPT9 (sh1, sh2) and SEPT2 (sh1, sh2) knockdown (Supplementary Table S2). As shown in Supplementary Fig. S2, SEPT9-sh1 and SEPT2-sh1 specifically downregulated the expression of SEPT9 and SEPT2, respectively. Accordingly, we generated the lentiviral expression vectors containing SEPT9-sh1, SEPT2-sh1, and scramble (non-silencing sequence) controls for gene knockdown experiments. Suppression of SEPT9 and SEPT2 was verified by qRT-PCR (Fig. 3a) and western blot (Figs. 3b, c). Although SEPT9-sh1 and SEPT2-sh1 alone achieved ~50% inhibition of their respective genes, the combination of SEPT9-sh1 and SEPT2-sh1 achieved ~90% inhibition of both SEPT9 and SEPT2, suggesting their synergistic effect in repression of gene expression (Fig. 3d).

### Suppression of SEPT9 and SEPT2 synergistically reduced GBM cell viability

**Fig. 4:**
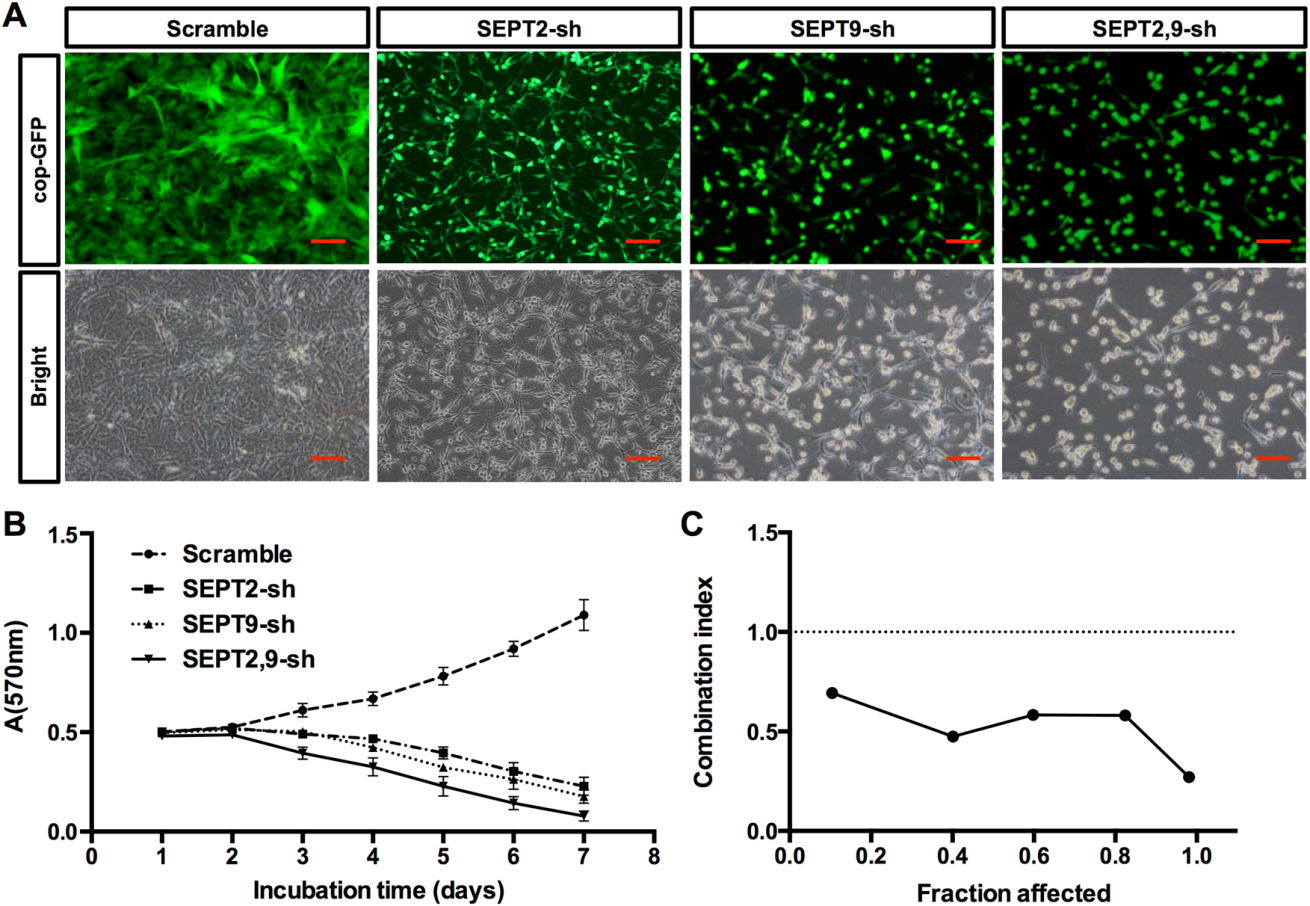
ShRNA-mediated suppression of A172 cell growth in vitro. **a** Scramble control A172 cells, SEPT2 and SEPT9 shRNA-transfected cells and SEPT2, 9 shRNA co-transfected cells (GFP is co-expressed) were photographed under fluorescence microscope, scale bar = 100 μm. **b** Cell viability was measured using the MTT assay. Cell growth curves were determined by reading the absorbance at 570 nm on a multiscanner reader. **c** The synergistic effect between SEPT2-sh and SEPT9-sh was shown as Fa-CI plot calculated with the CompuSyn software (CI < 1 indicates synergism, CI = 1 indicates an additive effect, and CI > 1 indicates antagonism)

The effects of SEPT9 and SEPT2 inhibition on GBM cell viability were examined in GBM cell line A172. Cells were transfected with the Scramble, SEPT2-sh, and SEPT9-sh expression vectors in which GFP is co-expressed (Fig. 4a). Along with Septin depletion and significant decrease in the number of cells, the shape of GBM cells changed remarkably as well. Scramble-treated cells seemed to have large cell bodies with long processes. SEPT2-sh-treated cells were relatively smaller and with thinner processes. Although SEPT9-sh and SEPT2, 9-sh-treated cells demonstrated a small and round shape (Fig. 4a). Furthermore, both SEPT9 and SEPT2 knockdown significantly inhibited A172 cell growth in a time-dependent manner (Fig. 4b and Supplementary Fig. S3A). Among the various groups of RNAi inhibitions, the SEPT9-sh and SEPT2-sh combination exerted the strongest inhibition of cell growth in A172 cells, revealing their synergistic inhibitory effect (CI was 0.27–0.69, CI < 1 indicates synergism) with a Fa value of 0.10–0.98 (Fig. 4c).

### SEPT9 and SEPT2 suppression arrested GBM cell cycle in the S phase

**Fig. 5:**
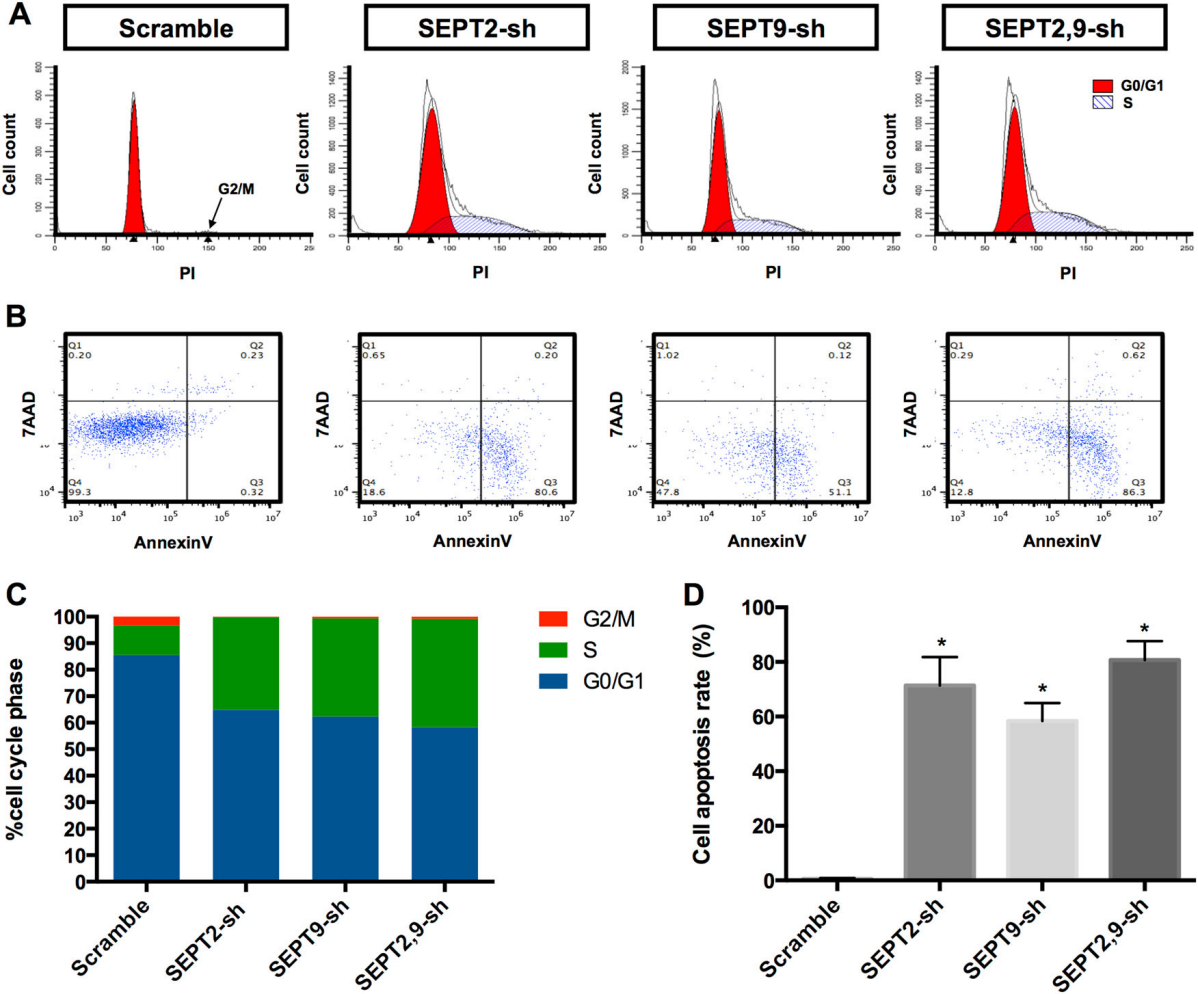
Effects of knocking-down SEPT2 and SEPT9 on A172 cell cycle progression and cell apoptosis. **a** Cell cycle progression detected by FACS analysis. Cells in G0/G1 were marked in the red area. Cells in S phase were marked with blue slash, whereas the arrowhead indicated the G2/M cells. **b** Cell apoptosis was assessed with Annexin V-7-AAD staining. **c** Cell cycle distribution was calculated with ModFit LT software. The combination of SETP2-sh and SEPT9-sh had the most S phase cell accumulation (40.9%). **d** The ratio of apoptotic cells in A172 cells treated with single shRNA was 71.4 or 58.4%, respectively, whereas the ratio of apoptotic cells in double shRNA-treated group was 80.7% (*, p<0.05).

After verifying the anti-proliferation effect of SEPT9-sh and SEPT2-sh, the distribution of cell cycles was explored by flow cytometry. As shown in Fig. 5a, the A172 cells in G0/G1 phase were decreased sharply in both SEPT9-sh and SEPT2-sh group (from 86% to around 63%), and there was no apparent difference between these two groups, although the combination group had the most reduction (from 86 to 58%) than the single treatment groups (Fig. 5c). As a result, the S phase cells accumulated, and the G2/M phase cells disappeared.

### Downregulation of SEPT9 and SEPT2 expression induced GBM cell apoptosis

To address whether the decreased cell number was attributable to apoptosis induced by SEPT9-sh and SEPT2-sh, we compared cell death in A172 cells treated with various inhibitory RNAs. Although the scramble RNA produced 0.6 ± 0.2% apoptotic cells, SEPT9-sh induced apoptosis in 58.4 ± 3.8% of A172 cells, SEPT2-sh induced 71.4 ± 6.0% and the combination of SEPT9-sh and SEPT2-sh yielded 80.7 ± 4.0% (dots in the lower right quadrant in Fig. 5b). Thus, knockdown of these two genes displayed a synergistic effect on inducing early apoptosis in A172 cells (Fig. 5d).

### SEPT9 and SEPT2 suppression synergistically inhibited migration and invasion of GBM cells in culture

**Fig. 6:**
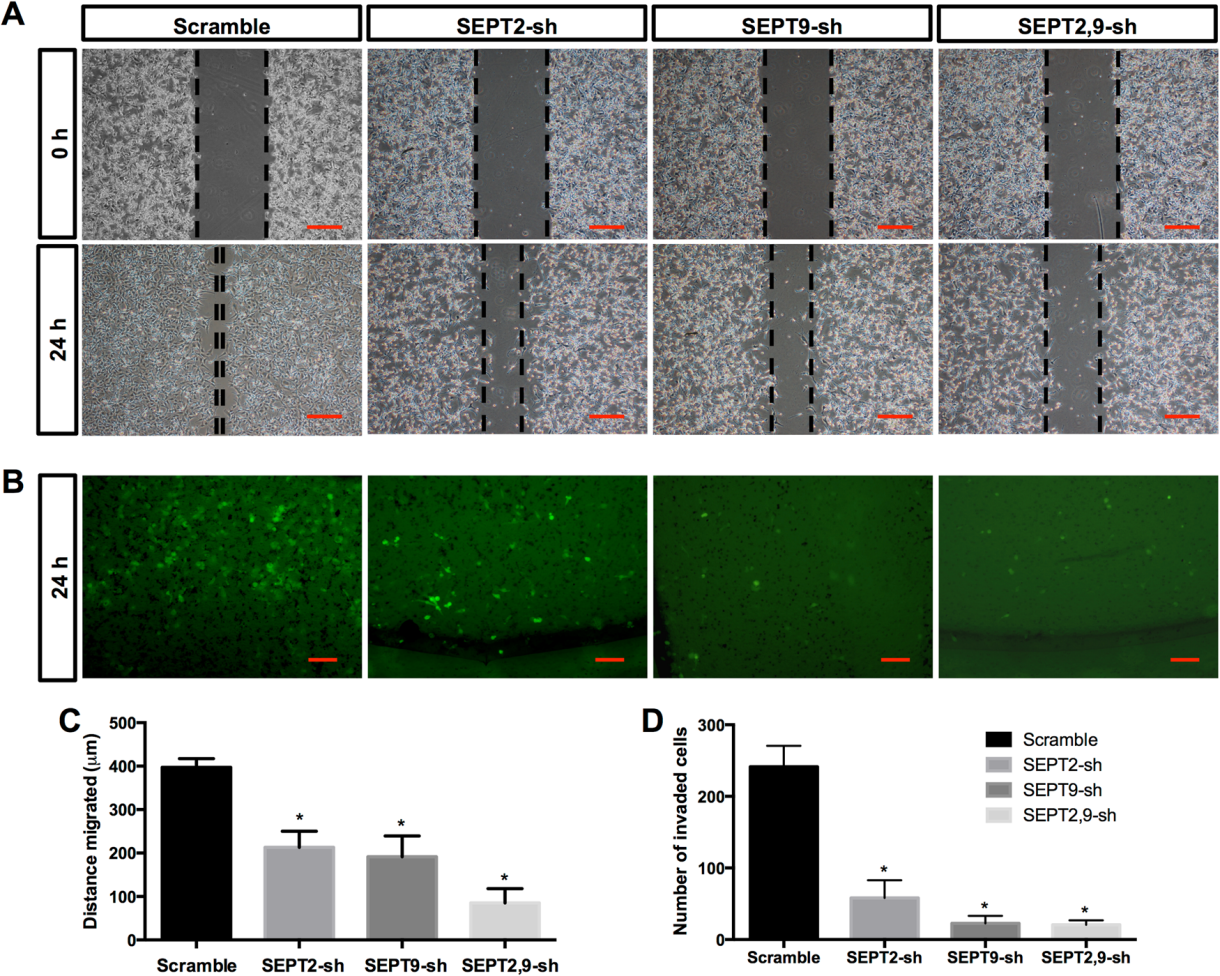
Suppression of SEPT2 and SEPT9 modulated A172 cell migration and invasion. **a** Effects of SEPT2 and SEPT9 knockdown on A172 cell migration, scale bar = 100 μm. **b** SEPT2 and SEPT9 shRNA diminished cell invasion of A172 cells (Transwell assay with 8-μm pore size), scale bar = 100 μm. **c** The migration distance of A172 cells was quantified by Image J software with the SEPT2, 9-sh group having the shortest migration distance (85 μm). **d** The mean cell counts of invading cells with the double knockdown group having the least invasion cells (around 21, *p < 0.05)

**Fig. 7:**
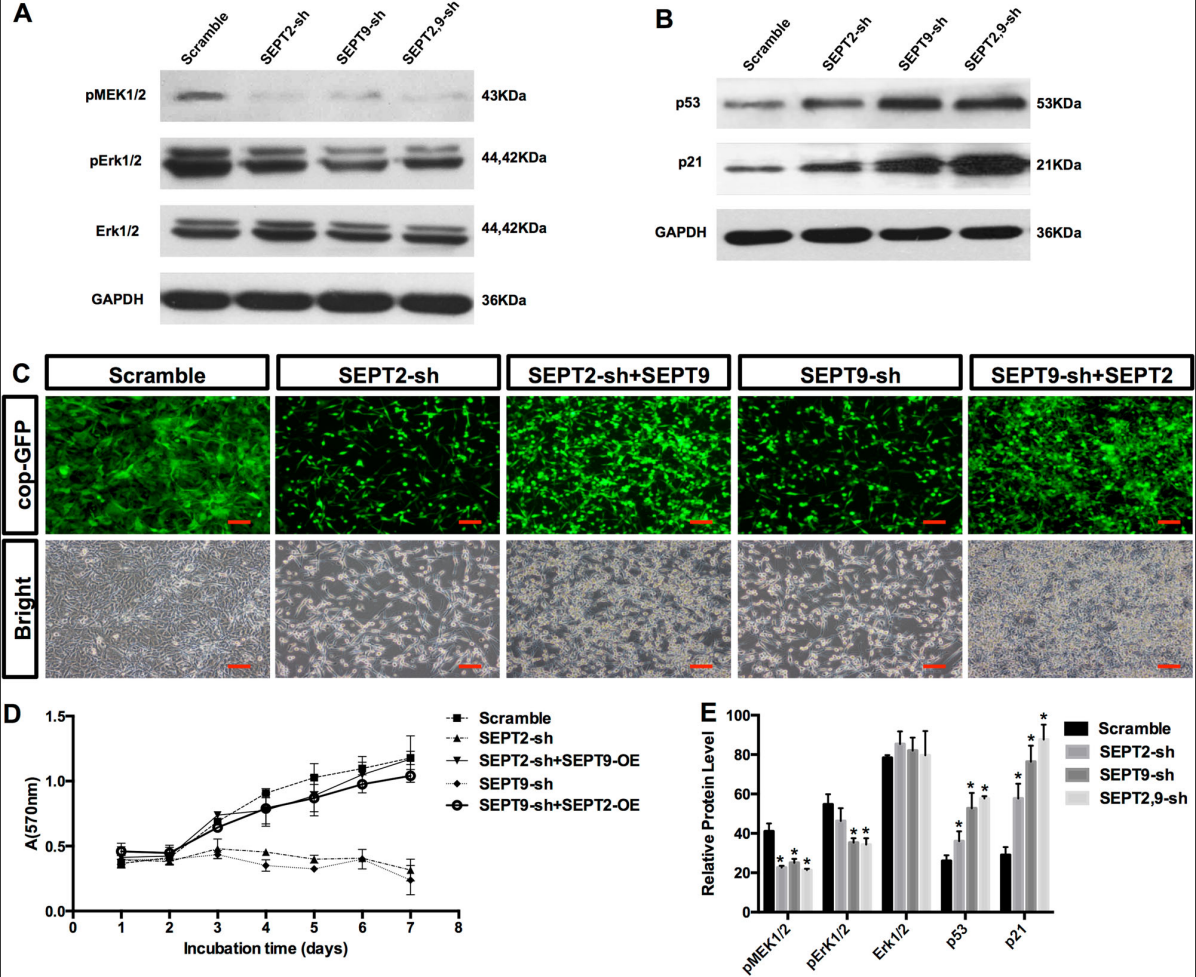
SEPT2 and SEPT9 shRNA inhibit GBM cell growth involving the MEK-ERK and p53-p21 pathways. **a** A decrease of pMEK1/2 and pErk1/2 protein levels in SEPT2 and SEPT9-depleted A172 cells. **b** An increase of p53 and p21 protein accumulation in SEPT2 and SEPT9-depleted A172 cells. **c** Overexpressing SEPT9 in SEPT2 depleted A172 cells rescued shRNA induced cell growth inhibition, and vice versa, scale bar = 100 μm. **d** Growth curves further indicated that overexpression of SEPT2 or SEPT9 reversed cell growth inhibitions by RNAi. **e** Quantitative analysis of western blot results from **a** and** b** (*p < 0.05)

We next examined the effects of SEPT9 and SEPT2 suppression on the 2D-migration and 3D-invasion of GBM cells by wound-healing assay (Fig. 6a) and transwell assay (Fig. 6b). Wound-healing involves a number of processes, including cell proliferation, migration, and the establishment of cell polarity. To limit the impact of cell growth on our wound-healing assay, we starved the cells before and during the wounding assay of the monolayer cells. Serum starvation can result in a reversible cell cycle arrest at the G0/G1 phase^46,47^, and consequently the inhibition of cell growth. Meanwhile, the vast majority of GBM cells did not start cell growth within 48 h after seeding into plate as judged by the growth curve (Figs. 4b and 7d and Fig. S3A). As wound-healing and invasion assays were performed within 24 h when the cells were still at the resting phase, the decrease in the diameter of a wound reflected the result of migration only. As shown in Fig. 6a, the migration distances were significantly decreased after shRNA treatment. The SEPT2, 9-sh group had the shortest migration distance, and no obvious difference in migration was noticed between SEPT9-sh and SEPT2-sh group (Fig. 6c). The combined group exhibited the least migration and invasion ability, which was further confirmed by transwell assay (Fig. 6b). As cell invasion is an important feature of GBM cells, the decreased invasive cell numbers (from around 240 to 21) through transwell chamber membranes indicated that shRNA treatment reduced not only the viability but also the motility of GBM cells (Fig. 6d).

### Suppression of SEPT9 and SEPT2 expression inhibited MEK-ERK activation and increased p53-p21 expression

A recent study has reported that activation of MEK-ERK, but not PI3K (Targeting the phosphoinositide 3-kinase)/AKT signaling pathway was correlated with the increased protein levels of SEPT2 and SEPT7 in breast cancer^29^. Therefore, we set to investigate the molecular mechanisms underlying the SEPT9/2 RNAi-induced anti-GBM effects. We observed that suppression of SEPT9 and SEPT2 specifically impaired MEK1/2 phosphorylation, and the phosphorylation of downstream Erk1/2 (Figs. 7a, e). There was no obvious increase in Akt activation, similar to the observation of SEPT2 and SEPT7 depletion in breast cancer cells (Supplementary Fig. S3B). This result implies that MEK-ERK axis might be pivotal to the functions of Septin family in different cancer cell types

As described earlier, SEPT9 and SEPT2 knockdown induced GBM cell cycle arrest in S phase (Figs. 5a, c) and massive cell apoptosis (Figs. 5b, d). Thus, we next examined the expression of cell cycle- and apoptosis-regulated protein p53 and p21. P53 protects mammals from neoplasia by inducing apoptosis, DNA repair, and cell cycle arrest in response to a variety of stresses^48^. As shown in Figs. 7b, e, p53 accumulated in single Septin knockdown groups and even more so in the double Septin knockdown group. Following p53 accumulation, the protein level of p21 was also upregulated, consistent with the previous observation that p53 could mediate the transcription of p21, which subsequently binds to the Cdc2-Cyclin B1 complex and inactivates it, leading to S phase cell cycle arrest.

### SEPT9 or SEPT2 overexpression rescued RNAi-induced cell growth inhibition

As SEPT9 and SEPT2 knockdowns suppressed the GBM cell growth in a synergistic manner, we speculate that overexpression of one Septin gene could compensate for the loss of another in GBM cells. Thus, we performed the rescue experiment in A172 and U87 cells. As expected, overexpression of SEPT9 in SEPT2 knocked-down cells restored the cell growth (Fig. 7c). Conversely, overexpression of SEPT2 in SEPT9-depleted cells had a similar effect in cell growth recovery (Fig. 7d). Interestingly, despite the full restoration of cell growth in both SEPT2 and SEPT9 rescued groups, the cells reshaped morphologically following RNAi treatments (Fig. 7c), suggesting that the effects of Septins on cell growth and cellular morphology depend on different mechanisms.

### Suppression of SEPT9 and SEPT2 inhibited GBM growth in vivo

**Fig. 8:**
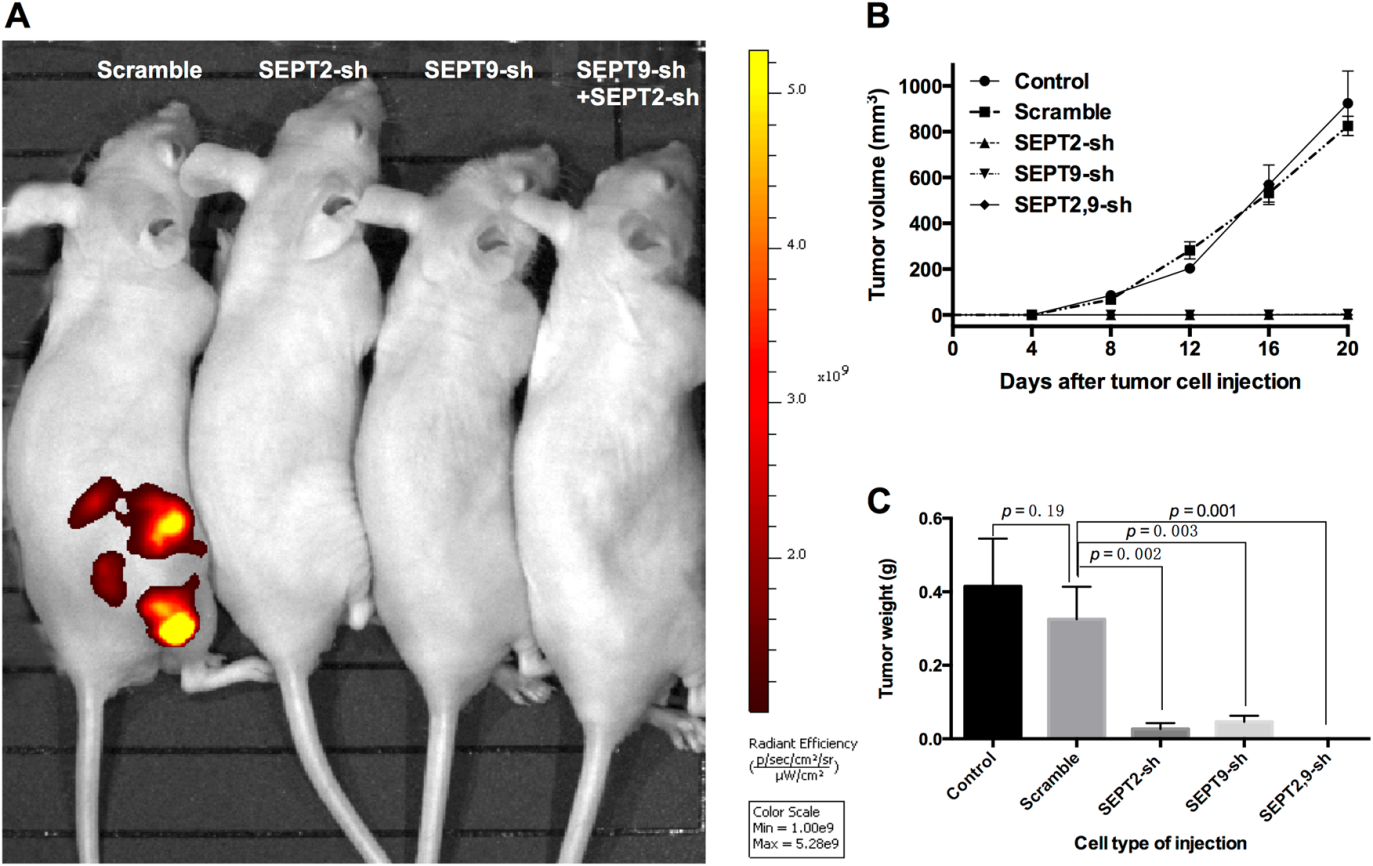
Effects of SEPT2 and SEPT9 knockdown on GBM cell-derived subcutaneous xenograft tumors in nude mice. **a** The in vivo bioluminescent imaging data were analyzed in different groups via the IVIS system. **b** Quantitative analyses of the tumor progression. Tumor size was determined by measuring the tumor volume every 4 days from day 4 to day 20 after injection. **c** Tumor weights in mice 20 days after injection. Both single and double shRNA-treated groups demonstrated significant decrease of tumor weights (*p* < 0.05)

To investigate the anti-GBM effect of SEPT9 and SEPT2 RNAi in vivo, we established a subcutaneous xenograft tumor model of GBM cells. After RNAi treatment, U87, U87-Scramble, U87-SEPT2-sh, U87-SEPT9-sh, and U87-SEPT2, 9-sh cells were injected into nude mice (Fig. 8a). Tumor volumes were measured at different time points of tumor growth in various groups (Fig. 8b). After 8 days, mice injected with U87-SEPT2-sh, U87-SEPT9-sh, and U87-SEPT2, 9-sh cells did not show any increase in mean tumor size as compared with the U87 and U87-Scramble groups. After 20 days, tumors in each mouse were removed and weighed. Compared with control and scramble groups, both single and double Septin RNAi treatments significantly decreased the solid tumor mass (Fig. 8c), indicating that downregulating the expression of SEPT9 and SEPT2 in GBM cells suppresses their tumor formation in vivo.

## Discussion

### Multi-omics approach increases confidence of SEPT9 and 2 as GBM associate genes

We have applied a multi-omics approach to developing a workflow from discovery of GBM-related candidates to validation of GBM functional molecules (Fig. 1). Through the integration of quantitative data from transcriptomics and proteomics, we have identified and validated SEPT9 and SEPT2 as potential targets for GBM treatment. SEPT9 and SEPT2 are two core elements of Septin family with similar cytosolic localization and biological functions (Fig. 2b), and have been implicated in a variety of human pathological conditions, including bacterial infection, Alzheimer disease, Parkinson disease, and male infertility^49^. *SEPT9* was identified as an oncogene in ovarian, head and neck, and prostate cancer cells^50–52^. Moreover, promoter methylation of SEPT9 was considered as a specific and reliable biomarker for the early detection of colorectal cancer^53^. SEPT2 downregulation was shown to suppress hepatoma and breast cancer cell growth^29,36^. Our study systematically investigated the functions of SEPT9 and SEPT2 in GBM tumorgenesis including tumor growth and invasion both in vitro and in vivo.

### Combinatorial strategy with SEPT9 and 2 RNAi facilitates GBM therapy

It is well established that combinatorial therapies consisting of anticancer drugs with different molecular targets result in synergistic effect that is generally more effective than monotherapy. Our findings that SEPT9 and SEPT2 shRNA synergistically restrained malignant behavior of GBM cells shed light on developing novel precision treatment of GBM with combinatorial RNAi. More importantly, we found that knockdown of SEPT9 and SEPT2 in normal human HDF cells did not disturb cell growth at all (Supplementary Fig. S3C), suggesting that SEPT9 and SEPT2 might have distinct functions in normal cell growth vs. tumor cell growth. It also implies that silencing SEPT9 and SEPT2 expression would be sufficient for GBM suppression with a minimum side effect.

### SEPT9 and 2 play roles opposite to SEPT7 in GBM cells

Previous studies showed that overexpression of SEPT7 could suppress glioma cell growth and induce cell cycle arresting in the G0/G1 phase^54–56^. In contrast, our results indicate downregulation of SETP9 or SEPT2 inhibits GBM cell proliferation and arrests cell cycle in S phase, suggesting the delicate and complex functional relationship among these three Septins in GBM. One key characteristic of Septins is their heterophilic interaction to form stable complex^18,24^. For example, SEPT9 can bind to SEPT2 and SEPT7 in a non­stoichiometric manner and stabilize the formation of higher­order complexes^57^. In support, we found that the silenced SEPT9 could slightly reduce the protein level of SEPT2, and vice versa (Figs. 3b, c). It is conceivable that the higher-order complex of SEPT9 and SEPT2 were able to protect or stabilize the single one from degradation^58^. This is also consistent with previous reports that knockdown of one Septin affects the protein level of another Septin from a different Septin subgroup^59,60^.

### The synergistic function of SEPT9 and 2 in GBM cells may involve two parallel pathways

From the perspective of downstream signal molecules, our findings demonstrate that knocking-down SEPT9 and SEPT2 synergistically upregulates the expression of p53 and p21 (Figs. 7b, e), which coordinate DNA repair, cell cycle control, or apoptosis initiation. It is not clear whether SEPT9 and SEPT2 could act on p53/p21 pathway directly or through an intermediate factor. It was reported that overexpression of SEPT7 inhibits glioma cell proliferation and arrests cell cycle progression by upregulation of p21^54^. It is plausible that SEPT9–SEPT7–SEPT2 complex might arrest SEPT7 and suppress its upregulation of p21 expression, and subsequently promote tumor growth. This might also explain the synergistic effect of silenced SEPT9 and SEPT2 simultaneously in GBM.

Our results also showed that knockdown of SEPT9 or SEPT2 in GBM cells reduces the activation of MEK/ERK pathway (Figs. 7a, e), which contributes to GBM cell proliferation, migration, invasion, and tumor formation in vivo^61^. Although MEK1/2 phosphorylation was impaired, levels of phospho-Akt stimulated by the activation of PI3K/AKT pathway remained the same (Fig. S3B). These results suggest that both SEPT9 and SEPT2 promote the GBM malignancy by activating the MEK/ERK, but not the PI3K/AKT pathway. Therefore, two parallel pathways (p53/p21 and MEK/ERK) are likely to be involved in the SEPT9 and SEPT2 regulation of GBM cell proliferation. Considering that Septins are cytoskeletal proteins^19,27^, it is also conceivable that they enhance GBM migration and invasion by interacting with actin, tubulin and myosin. In support, knocking-down SEPT9 and SEPT2 not only reduced the motility of GBM cells but also reshaped the cells morphologically (Fig. 4a).

### Possible compensatory mechanisms in SEPT2 and SEPT9

Our results showed that SEPT2 and SEPT9 could compensate each other in cell growth rescue experiments (Figs. 7c, d). The compensatory mechanisms for SEPT2 and SEPT9 are not known at this stage. It has been suggested that four Septin family members (SEPT2, 3, 6, 7) can form typical heterohexamer SEPT7-6-2-2-6-7 or hetero-octamer SEPT9-7-6-2-2-6-7-9, which then form higher-order structures such as filaments and rings^19^. In view of this finding, it is possible that overexpression of SEPT2 in SEPT9 knocked-down cells might increase the production of heterohexamers SEPT7-6-2-2-6-7, which can compensate for the loss of SEPT9. Recently, Kuo et al. reported that SEPT4 could occupy the same position as SEPT2^62^, and therefore overexpression of SEPT9 in SEPT2 knocked-down cells might induce the expression of SEPT4, and compensate for the loss of SEPT2.

## Conclusion

In summary, our study demonstrated that SEPT9 and SEPT2 are essential for GBM cell proliferation, migration, and invasion by controlling MEK/ERK activation and p53/p21 expression. SEPT9 and SEPT2 knockdown by RNAi in GBM cells exerts a synergistic antitumor effect. These findings suggest that the Septin proteins might be novel targets for GBM treatment.

## Electronic supplementary material

Tables

FigureS1

FigureS2

FigureS3

Supplementary figure legends
