# Supplementary material for: Repression of Septin9 and Septin2 suppresses tumor growth of human glioblastoma cells
Source: Cell Death Dis. 2018 May 3;9(5):514. doi: 10.1038/s41419-018-0547-4 (PMC5938713; doi:10.1038/s41419-018-0547-4)
Supplement: Supplementary file 1 — Tables [file 41419_2018_547_MOESM1_ESM.pdf]

Table S1 Details of four previous GBM transcriptomic studies from GEO repository for the multiplex analysis.

GEO	Glioma types	Chip	Probs	Source	GBM	Normal	Contributors
GSE50161	Ependymomas, glioblastomas, medulloblastomas, pilocytic astrocytomas	HG-U133_Plus_2	54675	Homo sapiens	34	13	Griesinger AM et.al. J Immunol 2013
GSE15824	Glioblastomas, astrocytoma and oligodendrioglioma	HG-U133_Plus_2	54675	Homo sapiens	12	5	Morin PJ et.al. Cancer Res 2011
GSE16011	Oligodendrogliomas(II,III), astrocytomas(II,III), glioblastomas(IV), oligoastrocytomas(II,III), pilocytic astrocytomas(I)	HG-U133_Plus_2	54675	Homo sapiens	106	8	Gravendeel LA et.al. Cancer Res 2009
GSE4290	Astrocytomas(II,III), glioblastomas(IV),oligodendrogliomas(II,III)	HG-U133_Plus_2	54675	Homo sapiens	81	23	Sun L et.al. Cancer Cell 2006
Total					233	49	

Table S2 SEPT2 and SEPT9 shRNA sequences.

Gene	Sequence(5'-3')
SEPT2-sh1	GATCCGCTATGGTGACGCTATCAACTGCAGAGATTCAAGAGATCTCTGCAGTTGATAGCGTCACCATAGCTTTTTG<br>AATTCAAAAAGCTATGGTGACGCTATCAACTGCAGAGATCTCTTGAATCTCTGCAGTTGATAGCGTCACCATAGCG
SEPT2-sh2	GATTCGGCAGGAAAGTGGAGAATGAGGACATGATCAAGAGTCATGTCCTCATTCTCCACTTTCCTGCCTTTTTG<br>AATTCAAAAAGGCAGGAAAGTGGAGAATGAGGACATGACTCTTGATCATGTCCTCATTCTCCACTTTCCTGCCG
SEPT9-sh1	GATCCGAGATCAAGTCCATCACGCACGATATTTCAAGAGAATATCGTGCGTGATGGACTTGATCTCTTTTTG<br>AATTCAAAAAGAGATCAAGTCCATCACGCACGATATTCTCTTGAAATATCGTGCGTGATGGACTTGATCTCG
SEPT9-sh2	GATCCGTGGTCAACATCGTCCCTGTCATTCAAGAGATGACAGGGACGATGTTGACCACTTTTTG<br>AATTCAAAAAGTGGTCAACATCGTCCCTGTCATCTCTTGAATGACAGGGACGATGTTGACCACG

Table S3 Primer sequences and information for SEPT2 and SEPT9 qRT-PCR analysis

Gene	Primers	Annealing temperature/^{0}C	Amplicon
Septin2	5’-TAAACAGCCTATTCCTAACT- 3’<br>5’-CATAAACGCCACATCTAA- 3’	50.8<br>49.66	344
Septin9	5’-TTATGAAACGCCTGAGCAAG- 3’<br>5’-GCCCACCACAGCAAATGG- 3’	56.43<br>59.34	218
GAPDH	5’-GGAGCGAGATCCCTCCAAAAT- 3’<br>5’-GGCTGTTGTCATACTTCTCATGG- 3’	61.6<br>60.9	197
