# Supplementary figures and images for: Repression of Septin9 and Septin2 suppresses tumor growth of human glioblastoma cells
Source: Cell Death Dis. 2018 May 3;9(5):514. doi: 10.1038/s41419-018-0547-4 (PMC5938713; doi:10.1038/s41419-018-0547-4)

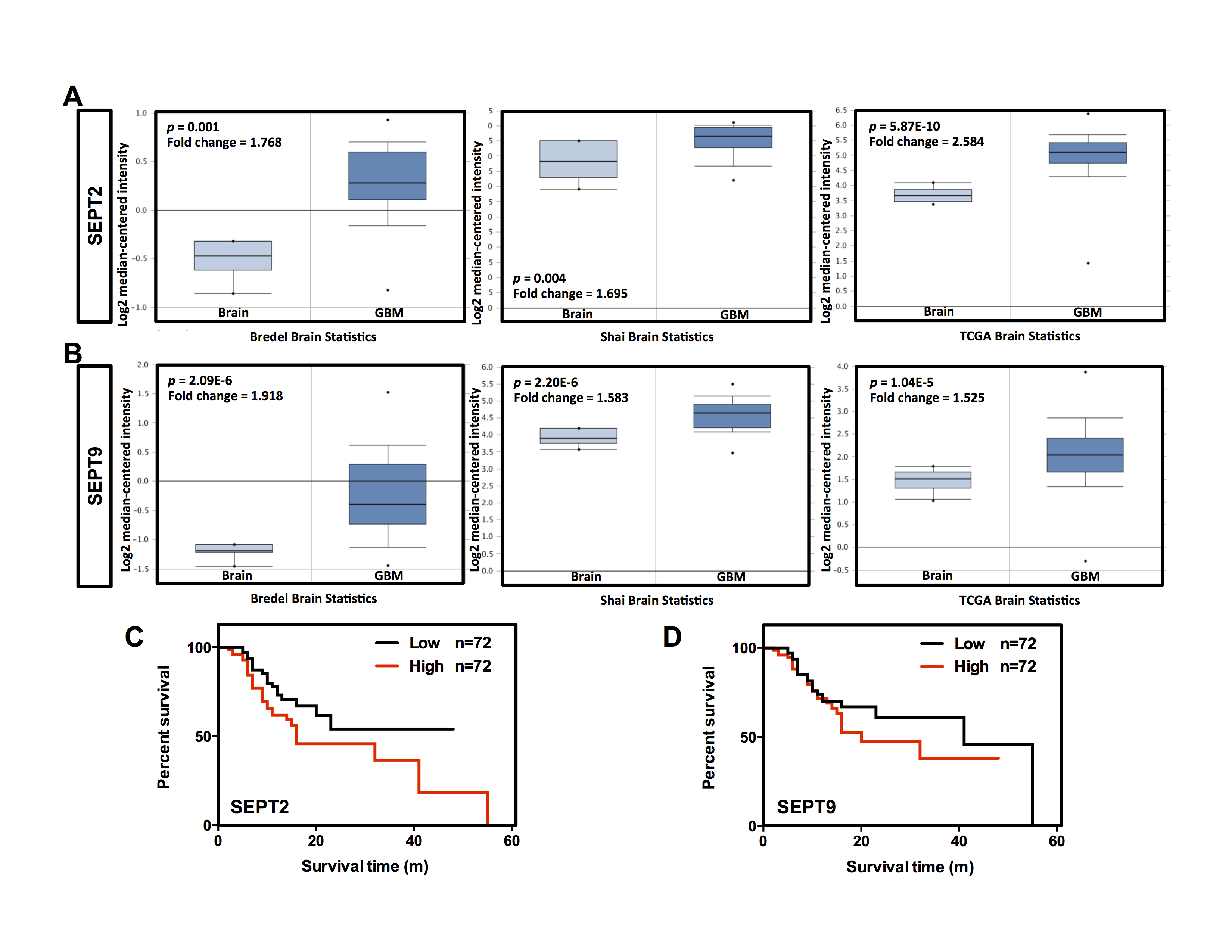

Supplement: Supplementary file 2 — FigureS1 [file 41419_2018_547_MOESM2_ESM.tif]

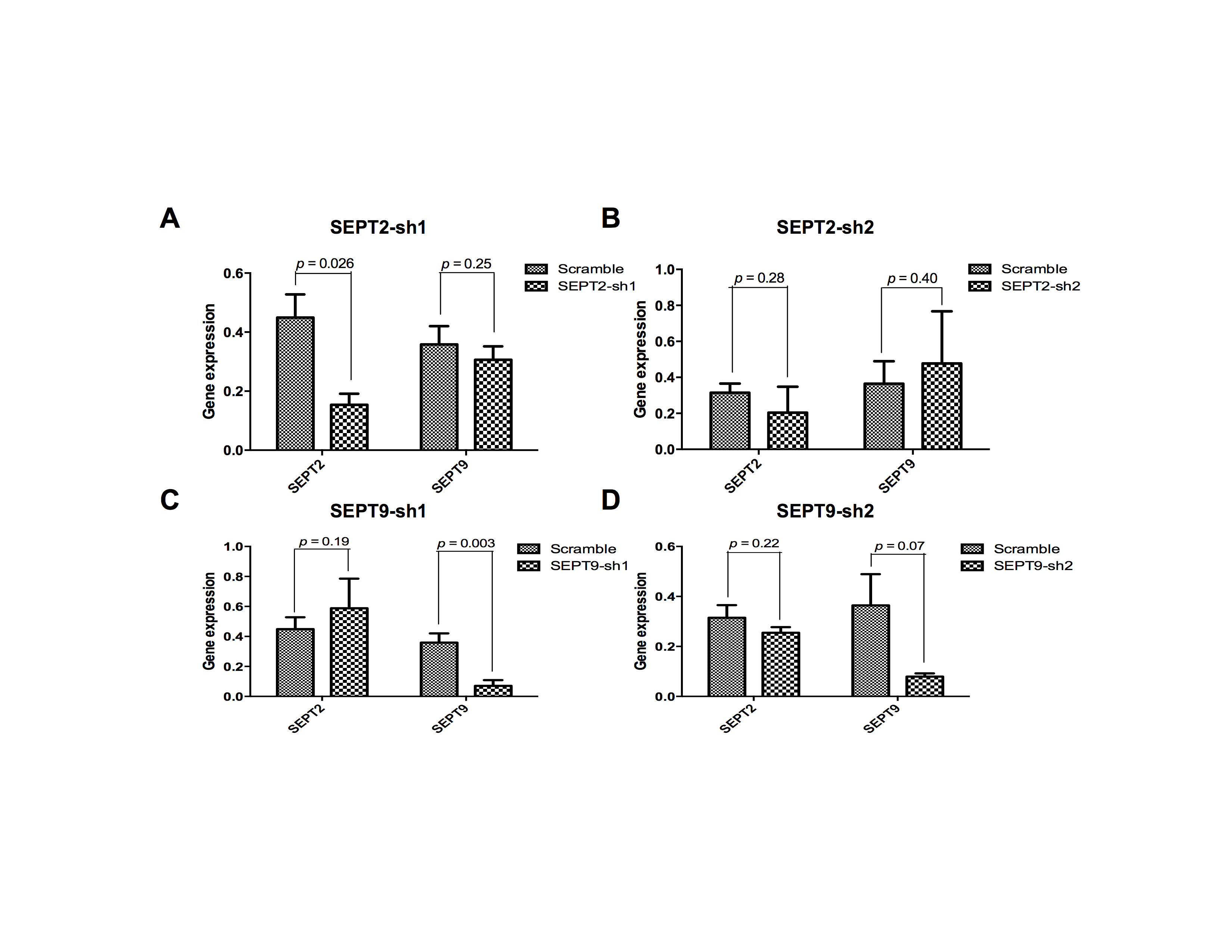

Supplement: Supplementary file 3 — FigureS2 [file 41419_2018_547_MOESM3_ESM.tif]

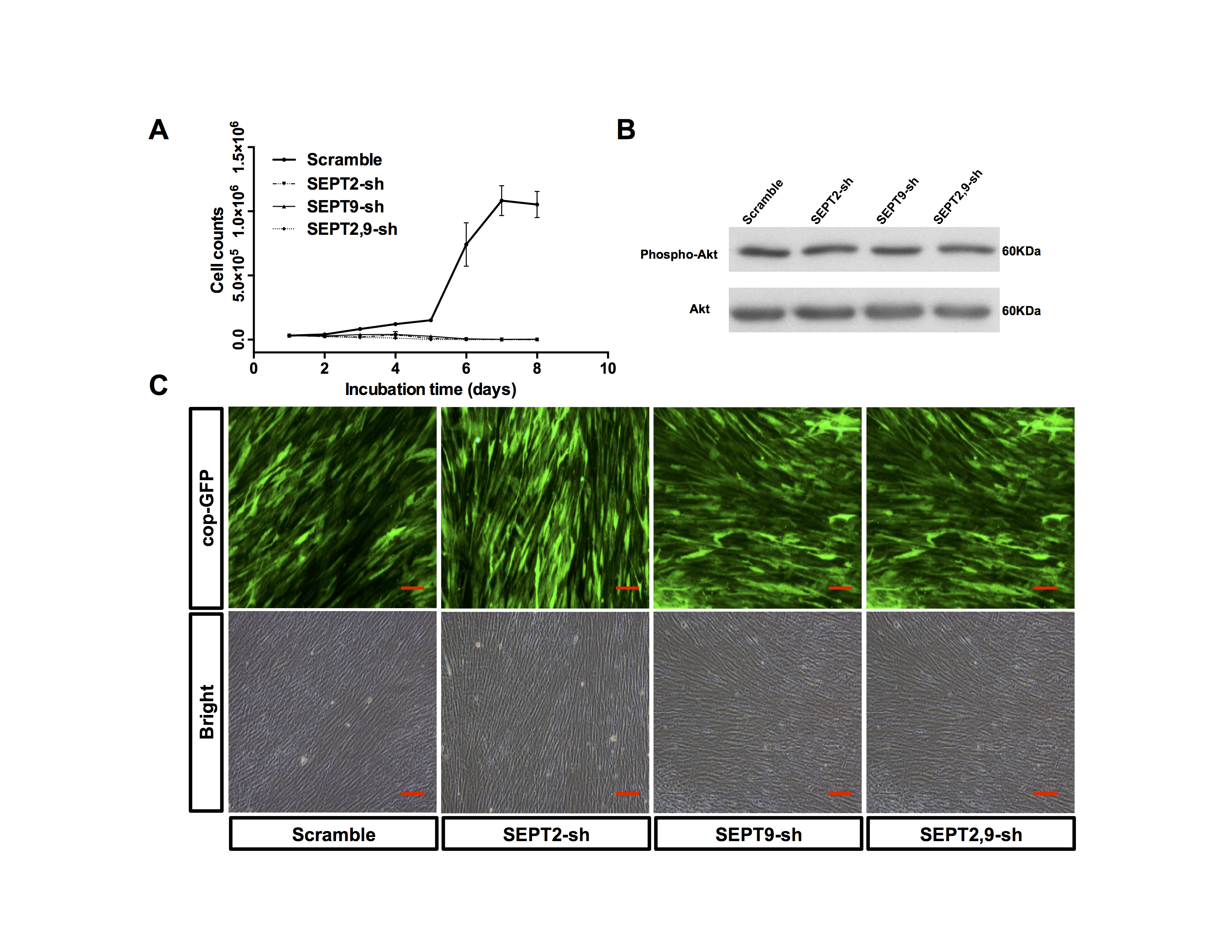

Supplement: Supplementary file 4 — FigureS3 [file 41419_2018_547_MOESM4_ESM.tif]
