# Supplementary material for: Repression of Septin9 and Septin2 suppresses tumor growth of human glioblastoma cells
Source: Cell Death Dis. 2018 May 3;9(5):514. doi: 10.1038/s41419-018-0547-4 (PMC5938713; doi:10.1038/s41419-018-0547-4)
Supplement: Supplementary file 5 — Supplementary figure legends [file 41419_2018_547_MOESM5_ESM.docx]

**Table S1 Details of four previous GBM transcriptomic studies from GEO repository for the multiplex analysis.**

**Table S2 SEPT2 and SEPT9 shRNA sequences.**

**Table S3 Primer sequences and information for SEPT2 and SEPT9 qRT-PCR analysis**

**Fig. S1 Increased SEPT2 and SEPT9 expression in GBM.** (A) Boxplots of SEPT2 expression levels in GBM and normal brain tissue samples in Bredel (27 GBM vs. 4 Normal), Shai (27 GBM vs. 7 Normal) and TCGA (542 GBM vs. 10 Normal) studies (GBM vs. Normal, *p*≤0.001 in all 3 studies). (B) Boxplots of SEPT9 expression levels in GBM and normal brain tissue samples in Bredel, Shai and TCGA studies (GBM vs. Normal, *p*＜0.001 in all 3 studies). Kaplan-Meier survival curve indicated higher SEPT2 (C) or SEPT9 (D) expression was unfavorable for patient survival.

**Fig. S2 Screening of effective shRNA targeting SEPT2 and SEPT9.** (A, B) In the SEPT2 RNA interference group, the SEPT2-sh1 specifically down-regulated the expression of SEPT2 by 66.7% (*p*=0.026), while the expression change of SEPT9 was statistically insignificant (*p*=0.25). (C, D) In the SEPT9 RNA interference group, the SEPT9-sh1 specifically down-regulated the expression of SEPT9 by 80.6% (*p*=0.003) and the expression of SEPT2 showed insignificant change (*p*=0.19).

**Fig. S3 Knocking-down SEPT2 and SEPT9 expression inhibited the growth of GBM cells, but not normal human cells.** (A) Growth curves of A172 cells under different conditions. (B) Levels of phospho-AKT remained unchanged, which indicated that PI3K/AKT signaling pathway was not involved in cell growth inhibition of silenced SEPT2 and SEPT9 in A172 cells. (C) Knockdown of SEPT9 and SEPT2 in normal human HDF cells did not disturb the HDF cell growth, scale bar=100 μm.
